# Cancer Diagnosis Using Deep Learning: A Bibliographic Review

**DOI:** 10.3390/cancers11091235

**Published:** 2019-08-23

**Authors:** Khushboo Munir, Hassan Elahi, Afsheen Ayub, Fabrizio Frezza, Antonello Rizzi

**Affiliations:** 1 Department of Information Engineering, Electronics and Telecommunications (DIET), Sapienza University of Rome, Via Eudossiana 18, 00184 Rome, Italy; 2 Department of Mechanical and Aerospace Engineering (DIMA), Sapienza University of Rome, Via Eudossiana 18, 00184 Rome, Italy; 3 Department of Basic and Applied Science for Engineering (SBAI), Sapienza University of Rome, Via Antonio Scarpa 14/16, 00161 Rome, Italy

**Keywords:** deep learning, convolutional neural networks (CNNs), generative adversarial models (GANs), deep autoencoders (DANs), restricted Boltzmann’s machine (RBM), recurrent neural networks (RNNs), long short-term memory (LTSM)

## Abstract

In this paper, we first describe the basics of the field of cancer diagnosis, which includes steps of cancer diagnosis followed by the typical classification methods used by doctors, providing a historical idea of cancer classification techniques to the readers. These methods include Asymmetry, Border, Color and Diameter (ABCD) method, seven-point detection method, Menzies method, and pattern analysis. They are used regularly by doctors for cancer diagnosis, although they are not considered very efficient for obtaining better performance. Moreover, considering all types of audience, the basic evaluation criteria are also discussed. The criteria include the receiver operating characteristic curve (ROC curve), Area under the ROC curve (AUC), F1 score, accuracy, specificity, sensitivity, precision, dice-coefficient, average accuracy, and Jaccard index. Previously used methods are considered inefficient, asking for better and smarter methods for cancer diagnosis. Artificial intelligence and cancer diagnosis are gaining attention as a way to define better diagnostic tools. In particular, deep neural networks can be successfully used for intelligent image analysis. The basic framework of how this machine learning works on medical imaging is provided in this study, i.e., pre-processing, image segmentation and post-processing. The second part of this manuscript describes the different deep learning techniques, such as convolutional neural networks (CNNs), generative adversarial models (GANs), deep autoencoders (DANs), restricted Boltzmann’s machine (RBM), stacked autoencoders (SAE), convolutional autoencoders (CAE), recurrent neural networks (RNNs), long short-term memory (LTSM), multi-scale convolutional neural network (M-CNN), multi-instance learning convolutional neural network (MIL-CNN). For each technique, we provide Python codes, to allow interested readers to experiment with the cited algorithms on their own diagnostic problems. The third part of this manuscript compiles the successfully applied deep learning models for different types of cancers. Considering the length of the manuscript, we restrict ourselves to the discussion of breast cancer, lung cancer, brain cancer, and skin cancer. The purpose of this bibliographic review is to provide researchers opting to work in implementing deep learning and artificial neural networks for cancer diagnosis a knowledge from scratch of the state-of-the-art achievements.

## 1. Introduction

Cancer is the leading cause of deaths worldwide [1]. Both researchers and doctors are facing the challenges of fighting cancer [2]. According to the American cancer society, 96,480 deaths are expected due to skin cancer, 142,670 from lung cancer, 42,260 from breast cancer, 31,620 from prostate cancer, and 17,760 deaths from brain cancer in 2019 (American Cancer Society, new cancer release report 2019) [3]. Early detection of cancer is the top priority for saving the lives of many. Typically, visual examination and manual techniques are used for these types of a cancer diagnosis. This manual interpretation of medical images demands high time consumption and is highly prone to mistakes.

For this reason, in the early 1980s [4], computer-aided diagnosis (CAD) systems were brought to assist doctors to improve the efficiency of medical image interpretation. Feature extraction is the key step to adopt machine learning. Different methods of feature extraction for different types of cancer have been investigated in [5,6,7,8,9,10,11,12,13,14,15,16,17,18,19,20,21]. However, these methods based on feature extraction have weaknesses. To overcome these weaknesses and to enhance the performance, representation learning has been proposed in [22,23]. Deep learning has the advantage of generating directly from raw images the high-level feature representation. In addition to deep learning, Graphics Processing Units (GPU) are also being used in parallel, for feature extraction and image recognition. For example, convolutional neural networks have been able to detect cancer with promising performance [24].

To test these algorithms, there are publicly available datasets. These include INbreast and BreakHis for breast cancer testing; Digital Database for Screening Mammography (DDSM)for mass detection; MITOSTAPIA for mitosis detection; Japenese Society of Radiological Technology (JSRT), The Lung Image Database Consortium (LIDC) and Image Database Resource Initiative (IDRI), and Danish Lung Cancer Screening Trial (DLCST) for lung nodule classification; multimodal Brain Tumor Segmentation challenge (BraTS) for brain cancer identification; and Dermoscopic Image Segmentation (DermIS) as well as data given to the public by International Skin Image Collaboration (ISIC) for skin cancers.

## 2. Steps of Cancer Diagnosis

### 2.1. Pre-Processing

Raw images contain noise in it so the first step in detection procedure is preprocessing, i.e., improving the quality of an image to be used further by the removal of unwanted image information, which is referred to as the image noises. Several inaccuracies may occur in the classification if this issue is not entertained properly. In addition to inaccuracies, the requirement of performing this preprocessing is because of low contrast among skin lesion and surrounding healthy skin, irregular border and the skin artifacts, which are hairs, skin lines, and black frames. Many filters can be applied for removal of Gaussian noise, speckle noise, Poisson noise, and salt and pepper noise, including median filter, mean filter, adaptive median filter, Gaussian filter, and adaptive wiener filter. For example, an image containing hairs in it along with the lesion may cause misclassification.

The image noises are supposed to be removed or adjusted by performing pre-processing tasks such as contrast adjustment, vignetting effect removal, color correction, image smoothing, hair removal, normalization, and localization. The right combination of pre-processing tasks gives more accuracy. Some of the preprocessing techniques are black frame removal techniques, automatic color equalization, hair removal technique, dull Razor, Karhunen–Loe’ve transform [25], Gaussian filter, pseudo-random filter, non-skin masking, color space transform, and contrast enhancement. The MRI images of brain cancer are at first converted into greyscale and then undergo contrast adjustment using smoothing operation [26]. Skull stripping is also performed on brain MRI images using a brain extraction tool (BET) and the extraction of brain tissues from other parts of skull [27]. Using X-ray machines, the computed tomographic (CT) images obtained for lung cancer diagnosis are preprocessed by first converting them into grayscale images, followed by the normalization procedure and noise reduction. These images are then converted into binary images, after which the unwanted part is removed [28]. Preprocessing in breast cancer particularly consists of delineation of tumors from the background, breast border extraction and pectoral muscle suppression. Mammograms, which are used for breast cancer diagnosis, include many noises, which are the high-intensity rectangular label, low-intensity label, and tape artifacts. Thus, mammogram labeling, orientation, and segmentation are done using preprocessing [29]. For prostate cancer diagnosis, transrectal ultrasound (TRUS) images are obtained, which have inherent noise and low resolution of images. The preprocessing module used for the noise suppression and artifacts consists of: (a) tree-structured nonlinear filtering (TSF); (b) directional wavelet transform (DWT); and (c) tree-structured wavelet transform (TSWT) [30].

### 2.2. Image Segmentation

Division of the input image into regions where the necessary information for further processing can be extracted is known as segmentation. Segmentation is basically the separation of a region of interest (ROI) from the background of the image. ROI is the part of the image that we want to use. In the case of cancerous images, we need the lesion part to extract the features from the diseased part. Segmentation can be divided into four main classes: (i) threshold-based segmentation; (ii) region-based segmentation; (iii) pixel-based segmentation; and (iv) model-based segmentation. Threshold-based segmentation includes Ostu’s method, maximum entropy, local and global thresholding, and histogram-based thresholding. Watershed segmentation and seeded region growing are examples of region-based segmentation. Fuzzy c-means clustering, artificial neural networks, and Markov field method are some of the methods of the class of pixel-based segmentation. Model-based segmentation is a parametric deformable model, e.g. level sets. There are many other methods for image segmentation: histogram thresholding, adaptive thresholding, gradient flow vector, distributed and localized region identification, clustering and statistical region growing [31], bootstrap learning [32], active contours, supervised learning, edge detection, fuzzy-C Mean clustering, probabilistic modeling, sparse coding [33], contextual hypergraph [34], cooperative neural network segmentation, principle component transform, and region fused band and narrow band graph partition [35], among others. Hybrid models of these methods by combining two or more have been used to improve the accuracy of the system.

### 2.3. Post-Processing

After passing through the stages of preprocessing and image segmentation, there awaits post-processing where the task is to grab features. To accomplish this, the most common post-processing methods are opening and closing operations, island removal, region merging, border expansion, and smoothing. Some techniques used for the feature extraction are: principle component analysis (PCA), wavelet Packet Transform (WPT) [36,37], grey level co-occurrence matrix (GLCM) [38], fourier power spectrum (FPS) [39], Gaussian derivative kernels [40], and decision boundary features [41]. The basic steps of cancer diagnosis are summarized in Table 1.

### 2.4. ABCD-Rule

**Figure 1:**
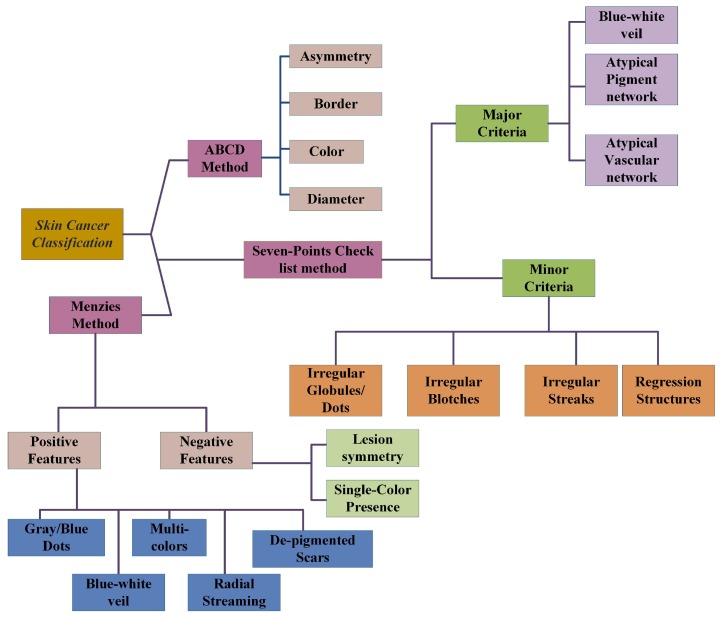
Summary of typically used skin cancer classification methods.

ABCD-rule analysis [42] refers to asymmetry (A), border (B), color (C) and diameter (D) of the lesion image). (A) Asymmetry: The input image is divided into a perpendicular axis in such a way that it gives the lowest possible value of asymmetry score. The score will be 2 if the asymmetry is with respect to the axes. If it is asymmetric on one axis, then its score will be 1. No asymmetry gives 0 scores. (B) Border: The image is divided into eight and checked for sharp and abrupt changes. Then, the score is checked, where a sharp cut off scores 1 and gradually scores 0. (C) Color: There are shades of colors for cancer detection: black and brown, but also sometimes white, red or pink. Colors are counted. (D) Diameter: The diameter of the lesion is carefully checked. If it is larger than 6 mm in diameter, then it is melanoma. Figure 1 shows the block diagram of all the methods described in this section.

### 2.5. Seven-Point Checklist Method

There are two types of criteria based on which classification is done. These are major and minor criteria. The major criteria have three points, and each point has a score value of 2, whereas minor criteria have four points each with a score value of 1. If the score value is at least 3, the classification result would be malignant melanoma [43].

#### 2.5.1. Major Criteria

**Blue-white veil:** These are blue blotches with a white haze around it having no defined structure.

**Atypical pigment network:** In this network, the lesion has asymmetric distribution within it along with reticular lines while the color and thickness are heterogeneous in nature.

**Atypical vascular pattern:** These are irregular globular or dotted vessels having linearity in it.

#### 2.5.2. Minor Criteria

**Irregular globules/dots:** Dots have an irregular shape, color, and distribution with size less than 0.1 mm, whereas globules size should be greater than 0.1 mm.

**Irregular blotches:** These are areas having different colors white, black or brown and no certain shape or regularity (no defined distribution or structure).

**Irregular Streaks:** When melanoma start growing radially, it forms radial streak type pattern and pseudopods, which are located at the edges of the lesion area.

**Regression structures:** These are scars such as de-pigmentation, particularly white in color.

### 2.6. Menzies Method

There are a few positive features (Pos.F) and negative features (Neg.F). The presence of any negatives declares melanoma to be malignant. It would be benign if both negatives are absent and one or more positives are true [43]. These are summarized in Table 2.

### 2.7. Pattern Analysis

There is a method based on finding patterns which are local or global. Global patterns can be homogenous, globular, starburst, reticular, parallel multi-component, or cobblestone. Local patterns can be the irregular steaks, inadequate pigmentation, pigment network, regression structures, globules, black dots, vascular structures or blue-white veil. The basis of this method is the qualitative assessment of the dermoscopic criteria individually.

## 3. Artificial Neural Networks

**Figure 2:**
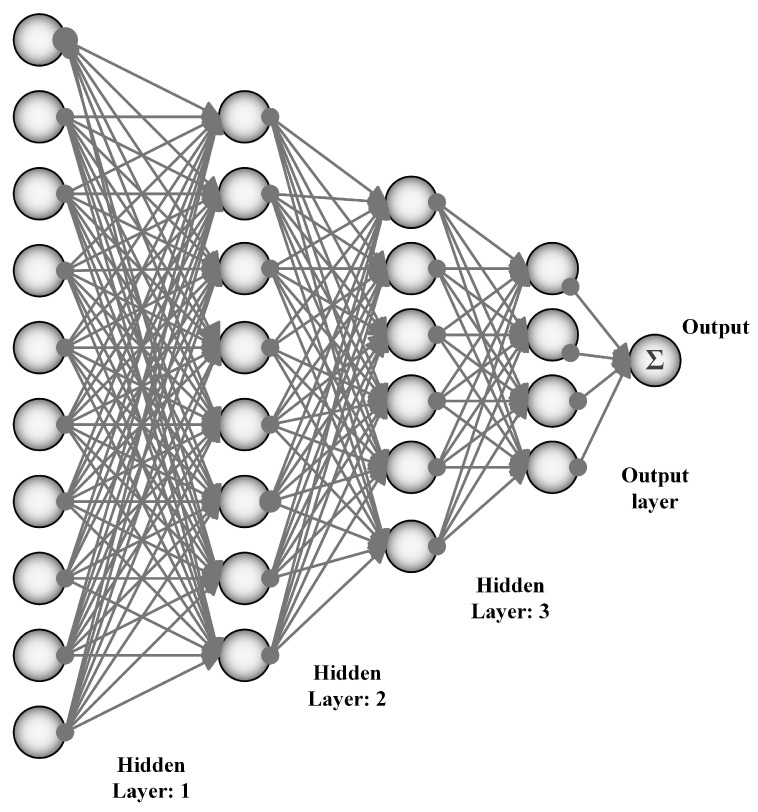
Artificial Neural Networks (ANNs).

Neural networks are capable of performing the tasks of complex computation because of the nonlinear processing of neurons. An artificial neural network is shown in Figure 2 As the artificial neural network has the power of prediction, it can be used for medical images. In a general artificial neural network, test images are given to the neurons for training. To train neurons, back-propagation algorithm is used, with the flow in the forward direction. Then, the generated output is matched with the desired output and the error signal is generated in the case the outputs do not match. This error propagates in the backward direction. Weights are adjusted for error reduction. This processing is repeated until the error becomes zero. There is a layered structure in the neural network with the number of interconnected nodes and an activation function among them. These activation functions are tangent hyperbolic function, sigmoid function, piece-wise linear function, and threshold function. Input patterns are presented to the network through an input layer, which then connects to the hidden layer, and this hidden layer connects to the output layer. Below, some of the ANNs are explained in detail and the links to their corresponding codes are provided in Table 3.

### 3.1. Convolutional Neural Networks

**Figure 3:**
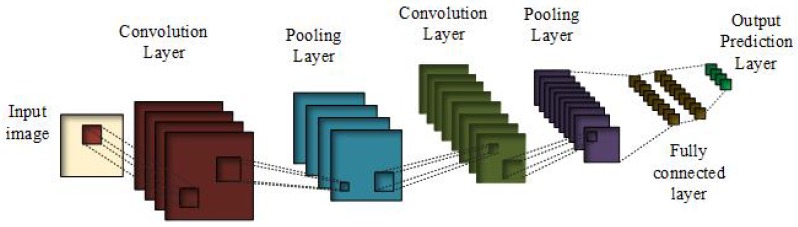
General convolutional neural networks.

CNN is a feed-forward neural network as shown in Figure 3. Here, the signal is processed directly without any loops or cycles. This can be represented as

(1) $$G\left(X\right)=g_{N}\left(g_{N-1}\left(...\left(g_{1}\left(X\right)\right)\right)\right)$$

where *N* represents number of hidden layers, *X* is the input signal and *g_N_* denotes the corresponding function to the layer *N*. A basic CNN model has a convolutional layer, which consists of a function *g* with multiple convolutional kernels *(h_1_, … h_k−1_, h_k_)*. Every *h_k_* denotes a linear function in *k*th kernel, represented as follows:

(2) $$h_{k}\left(x,y\right)=\sum_{s=-m}^{m}\sum_{t=-}^{n}\sum_{v=-d}^{w}Vk\left(s,t,v\right)X\left(x-s,y-t,z-v\right)$$

where *(x, y, z)* represents pixel position of input *X, m* represents height, *n* denotes width, *w* is depth of the filter, and *V_k_* represents weight of *k*th kernel.

The basic purpose of pooling in CNN is the task of subsampling i.e., it summarizes the nearby neighborhood pixels and replaces them in the output at a location with summarized characteristics. Pooling reduces the dimensionality and performs the invariance of rotational transformations and translation transformations. There are many pooling functions [44]; one of the most famous is max pooling, in which the output is the maximum value of the rectangular pixel neighborhood. In average pooling function, the output becomes the average of the rectangular neighborhood. Another type consists of the weighted average based on the distance from the central pixel. Pooling helps to make the representation invariant to small changes to the translation in the input.

Atrous Convolution is given by the following equation:

(3) $$y\left[i\right]=\sum_{k=1}^{K}x\left[i+r\cdot k\right]w\left[k\right]$$

where *x[i]* is the 1D input signal, *w[k]* is the filter of length of *k*, and *r* is the stride rate with which the input signal is sampled. *y[i]* is the output of the atrous convolution. Atrous convolution is applied over the input *x* for each location *i* on the output *y* and a filter *w* with the atrous rate *r*, which corresponds to the stride rate.

Deep residual learning is used to counter the degradation problem, which arises when the deep network starts to converge, i.e., a saturation of accuracy and degradation with the increasing depth. The residual network explicitly allows the stacked layers to fit in the residual map rather than a desired underlying map. According to the experimental results, the optimization of residual networks is easier, and the accuracy is achievable with a considerable increase in depth. Skip connections help the transverse information in deep neural networks. Due to passing through many layers, the gradient information may be lost, which is known as the vanishing gradients problem. Skip connection has the advantage of passing the feature information to lower layers, which makes it easier to classify the minute details. Some of the spatial information is lost due to the max-pooling operation, whereas skip connections make it possible to have more information on the final layer so that the classification accuracy increases.

In activation layer, different activation functions that can be used:

(i) Sigmoid activation function [45] is given by the equation:
  (4) $$\sigma \left(x\right)=\frac{1}{1+e^{-x}}$$
  It is nonlinear in nature; its combination will also be nonlinear in nature, which gives us the liberty to stack the layers together. Its range is from −2 to 2 on the x-axis and on y-axis it is fairly steep, which shows the sudden changes in the values of *y* with respect to small changes in the values of *x*. One of the advantages of this activation function is its output always remains within the range of (0,1).
(ii) Tanh function is defined as follows,
  (5) $$f\left(x\right)=\tanh\left(x\right)=\frac{2}{1+e^{-2x}}-1$$
  This is also known as the scaled sigmoid function:
  (6) tanh(x)=2sigmoid(2x)−1
  Its range is from −1 to 1. The gradient is stronger for the tanh than the sigmoid function.
(iii) Rectified linear unit (ReLU) is the most commonly used activation function [45,46,47], where *g* denotes pixel-wise function, which is nonlinear in nature. That is, it gives the output *x*, if *x* is positive and it is 0 otherwise.
  (7) g(x)=max(0,x)
  ReLU is nonlinear in nature and its combination is also nonlinear, meaning different layers can be stacked together. Its range is from 0 to infinity, meaning it can also blow up the activation. For the pooling layer, *g* reduces the size of the features while acting as a layer-wise down-sampling nonlinear function. A fully connected layer has a 1 × 1 convolutional kernel. Prediction layer has a softmax which predicts the probability belonging of *X_j_* to different possible classes.

### 3.2. Multi-Scale Convolutional Neural Network (M-CNN)

One of the multi-scale CNN architectures, as described by researchers in [48], consists of three convolutional blocks, each of which comprises a convolutional layer and a rectified linear unit (ReLU) followed by the max-pooling layer and two fully connected layers. Each input image is first down-sampled to multiple different scales and then the patches are collected. These patches are passed to the multi-scale CNN model scale-wise.

### 3.3. Multi-Instance Learning Convolutional Neural Network (MIL-CNN)

The problems in which the available labels are only for the set of data points are dealt with by the multi-instance learning MIL. Here, bags are used for the sets of data points and specific data points are referred to as instances. While using the binary labels, the most commonly made assumption is to consider a bag positive if at least one instance within the bag is positive. The mapping of instance space to the bag space has been made by using many functions, including Noisy-OR, generalization mean (GM), and log-sum-exponential (LSE).

#### CNN Architectures

Many CNN architectures have been proposed by several researchers in the past. They are briefly described in this section and the summary of CNN application are given in Table 4 and Table 5.

**(i)**  **LeNet-5**
  In 1998, a seven-level convolutional neural network was proposed, which was named as LeNet-5 by LeCun et al. The main advantage of this network was digit classification and was used by banks for the classification of handwritten numbers by costumers. They used 32 × 32 pixel grey-scale images as input for the classification. To process large images, high-resolution demands more convolutional layers, which limits this architecture.
**(ii)**  **AlexNet**
  AlexNet was a challenge winner architecture in 2012, by reducing the top-5 errors from 26% to 15.3%. This network is similar to LeNet but is deeper, with an increased number of filters per layer and more stacked convolutional layers. It consists of 11 × 11, 5 × 5, and 3 × 3 convolutional kernels, max pooling, dropout, data augmentation, and ReLU activations. ReLU activation is attached after every convolutional and fully connected layer. It takes two days to test this network on GPU580 Nvidia Geforce, which is why they split the network into two pipelines. The designers of ALexNet are a supervision group consisting of Alex Krizhevsky, Geoffrey Hinton, and Ilya Sutskever.
**(iii)**  **ZFNet**
  ZFNet was the winner of ImageNet Large Scale Visual Recognition Competition (ILSVRC) 2013. The authors reduced the top-5 error rate to 14.8%, which is half the non-neural error rate. They achieved it by keeping the AlexNet structure the same but changing its hyperparameters.
**(iv)**  **GoogleNet/ Inception V1**
  This was the winner of ILSVRC 2014 with the top-5 error rate of 6.67%, which is very close to human-level performance, thus the creators of the network were forced to perform human evaluation. After weeks of training, the human experts achieved top-5 error rate of 5.1% (single model) and 3.6% for ensemble. The network is a CNN based on LeNet dubbed with the inception module. It uses batch normalization, image distortions, and RMSprop. This is a 22-deep-layered CNN network but can reduce the parameters from 60 million to 4 million.
**(v)**  **VGGNet**
  VGGNet was the runner-up in ILSVRC 2014. It is made up of 16 convolutional layers and a uniform architecture. It has only 3 × 3 convolution but many filters. It was trained for three weeks on 4 GPUs. Because of its architectural uniformity, it is the most appealing network for the purpose of feature extraction from images. The weighted configurations of this architecture were made public and is has been used as the baseline for many applications and challenges as the feature extractor. The biggest challenge one faces for this network is its 138 million parameters, which become difficult to handle.
**(vi)**  **ResNet**
  Residual neural network (ResNet) at the ILSVRC 2015 uses skip connections and feature batch normalization. Those skip connections are also known as gated recurrent units, which are similar to the elements being applied recently in RNNS. This network-enables training a neural network with a 152 layers and a reduced complexity comparable to VGGNet. The achieved error rate of top-5 was 3.57%, thus it beats the human-level performance on the given dataset.

### 3.4. Fully Convolutional Networks (FCNs)

**Figure 4:**
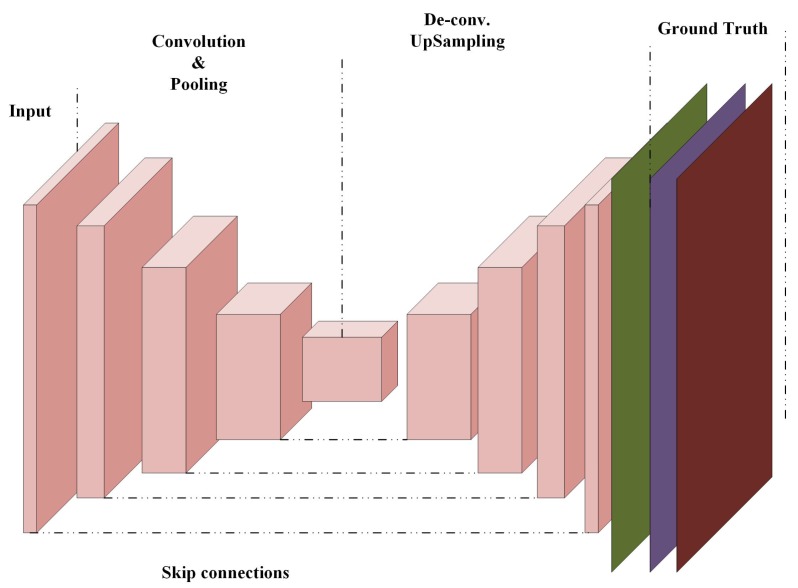
Fully convolutional neural networks.

FCN differs from CNN as in FCNs the fully connected layer is replaced by an up-sampling layer and a deconvolutional layer [106] as shown in Figure 4. These layers are considered to be the backward versions of pooling and convolutional layers, respectively. FCNs generate a score map for each class instead of generating one probability score. This map has the exact same size as the input image and classifies the image pixel by pixel. Then, accuracy is improved by using upsampling and deconvolutional layers (this is called skip connection). These new layers are used for the development of many deep learning algorithms in many applications [107,108,109].

#### 3.4.1. U-Net Fully Convolutional Neural Network

O.Ronneberger developed U-Net for biomedical image segmentation. Their architecture consists of two paths. The first contraction path is known as an encoder, which captures the context in the image. This is mainly a stack of convolution and pooling layers. The second path is the symmetric expanding path, also known as a decoder, which uses transposed convolutions and enables the precise localization. It is an end-to-end FCN network with no dense layer, only convolutional layers. Therefore, it can accept an image of any size.

#### 3.4.2. Generative Adversarial Networks (GANs)

Goodfellow et al. introduced a method named as a generative adversarial network GAN [110], which is basically a two-player min-max game having a generator as the first player and discriminatory as the second player. The transformation from the prior distribution $p_{z}$ of the random noise *z $∼p_{z}$* to realistically looking images *G(z) $∼p_{fake}$* is done by generative network *G*. [111]. Discriminator *D* network classifies the fake sample that generator *G(z)* generated, from the real training data distribution $x∼p_{real}$. Parameters of the generator *G* are adjusted using the feedback information from the discriminator *D* so that generators samples are able to fool the discriminator in the classification task. *D* produces better and more realistic fake samples while *G* learns and produces the real samples. GANs have the ability to map the random to a realistic distribution [112,113]. GANs have been used for various applications including reconstruction [114,115,116], segmentation [117,118,119], domain adaptation [120,121] and detection [122,123]. GANs have also been used for the synthetic data generation; for example, Hou et al. used nuclei masks dealing with the foreground and background separately to generate pathology data. For the 3D segmentation of images, the generator of GAN architecture is used to generated images from the learned data distribution $p_{data}\left(x\right)$ with the simultaneous training of the discriminator to differentiate between the generated images and true examples [124]. The generator maps the noise to the synthetic image vector. V.K.Singh [125] and his fellow researchers used conditional adversarial networks for the segmentation of breast mass from the mammography .

### 3.5. Recurrent Neural Networks (RNNs)

Recurrent neural networks are a powerful model of sequential data [126]. A hidden vector sequence *f = $\left(f_{1},\ldots ,f_{T}\right)$* is computed by the RNN from input sequence *v = $\left(v_{1},\ldots ,v_{T}\right)$* by iteration of *t* = 1 to *T* and an output sequence *o = $\left(o_{1},\ldots ,o_{T}\right)$* is obtained:

(8) $$f_{t}=F\left(W_{vf}v_{t}+W_{ff}f_{t-1}+b_{f}\right)$$

(9) $$o_{t}=W_{fo}f_{t}+b_{o}$$

where *W* denotes the weight matrix and *b* represents the bias vectors. *F* is the hidden layer function, which is usually sigmoid function. Recurrent neural networks are deep in time because they are a function of all the previous hidden states.

### 3.6. Long Short-Term Memory (LTSM)

Long short-term memory (LTSM) is a form of recurrent neural network [127] introduced by Hochreiter and Schmidhuber in 1997. The main purpose of designing the LTSMs was to avoid the long-term dependency problem. Remembrance of information is their default behavior. It has feedback connections, thus it is also referred to as a general-purpose computer. It has the ability to process sequences of data, for example, audio speech signal or video signals, along with single point dataset, i.e., images.

### 3.7. Restricted Boltzmann Machine (RBM)

The restricted Boltzmann machine is characterized by a very simple architecture. It is made up of a visible layer, which is also referred to as the input layer, and a hidden layer, arranged as a bipartite graph since there is intra-layer communication in RBM, which is the major restriction in this architecture. Restricted Boltzmann machines are trained to maximize the product of probabilities assigned to each pattern in a given training set, by a contrastive divergence algorithm performing Gibbs sampling.

### 3.8. Autoencoders (AEs)

**Figure 5:**
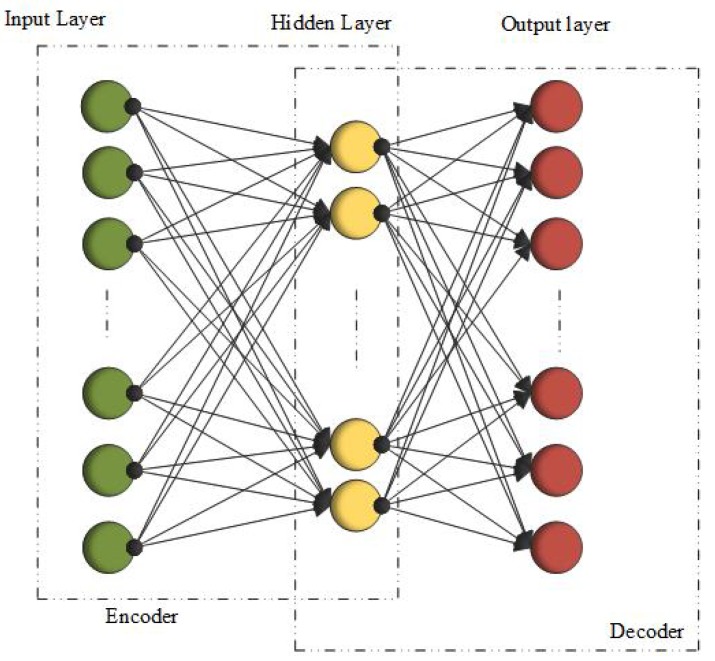
Autoencoders.

Autoencoders belong to the unsupervised learning class of neural networks [128]. A general example of auto-encoder is shown in Figure 5. They learn from the input data a lower dimensionality feature representation. The basic structure of AEs has an input layer followed by the hidden layer and an output layer [129]. Training is done through two stages: coding and decoding. In the first stage, input *I* is encoded by some representation *J* by some weight matrix $Y_{I,J}$ and bias $B_{I,J}$:

(10) $$J=\sigma (Y_{I,J}I+B_{I,J})$$

where σ is an activation function, also known as sigmoid function as given in (10)

In the second step, the representation J is decoded using new weight matrix $Y_{J,\hat{I}}$ and bias $B_{J,\hat{I}}$ to reconstruct $\hat{I}$:

(11) $$\hat{I}=\sigma^{\prime }\left(Y_{J,\hat{I}}+B_{J,\hat{I}}\right)$$

where $\sigma^{\prime }$ is also an activation function. $Y_{J,\hat{I}}$ can be considered as the transpose of $Y_{I,J}$ or new learnable matrix. These AEs are trained to minimize the error defined as:

(12) $$\arg\max_{Y,B}{∥I-\hat{I}∥}^{2}$$

### 3.9. Stacked Autoencoders

Stacking of *n* autoencoders into *n* hidden layers using unsupervised layer-wise learning followed by the fine-tuning using a supervised method makes the basic structure of the stacked autoencoders (SAEs) [130]. Hence, the SAE method is composed of three steps: Firstly, using input data, the first autoencoder is trained and the feature vector is formed. Secondly, this feature vector is the input of the next layer and the process is repeated until the end of the training of hidden layers. Third, a backpropagation (BP) scheme is used for minimization of the cost function after the training of the hidden layers and the weights are updated with labeled training set to obtain the fine-tuning.

### 3.10. Sparse Autoencoders SAE

These are a special type of autoencoders in which sparsity is introduced in the hidden units of the hidden layer by making the number of nodes in a hidden layer larger than the input layer. Stack of SAE (SSAE) is trained in greedy fashion while they are connected with the encoding part only. First, the hidden layer is separately trained as SAE, and the output of this layer becomes the input of the next layer training. Features are extracted by using low-level SAE, after which multiple SAE are stacked together where these extracted features are fed to the input of high-level SAE for the extraction of deeper features. Hence, these SSAEs are able to extract the deeper features from the data. A fully unsupervised sparse convolutional autoencoder (CAE) for the detection of the nucleus and for feature extraction of tissues from histopathology images was proposed by the researchers in [131]. The CAE network is composed of six convolutional layers and two average-pooling layers. Then, the network is divided into three branches: the nucleus detection branch, the foreground feature branch, and the background branch. The reconstructed images of foreground and background are made by decoding the foreground and background feature maps. The final image is constructed by adding the two intermediate images. The authors evaluated their method on four datasets and they could reduce the state-of-the-art system errors by up to 42%.

### 3.11. Convolutional Autoencoders CAE

Convolutional autoencoders learn features from the unlabeled images by using end-to-end learning scheme [132]. The spatial relationship between the image pixels makes it superior to the stacked autoencoders. It belongs to the category of unsupervised learning algorithms. Features can be extracted from them once the filters have been learned. The extracted features can easily be used to reconstruct the input. In CAEs, the number of parameters required to create an activation map is always the same, which makes it well suited for the scaled high dimensional images. If the fully connected layers of a simple autoencoder are replaced by the convolutional layer, it becomes the convolutional autoencoder. The sizes of the input layer and the output layer remain the same as in the simple autoencoder, except the decoding part, which changes to the convolutional network [133]. A self-clustering adversarial convolutional network with an unsupervised principle was proposed for the classification of prostate tissue as a tumor or non-tumor without the labeled data.

### 3.12. Deep Belief Networks (DBN)

**Figure 6:**
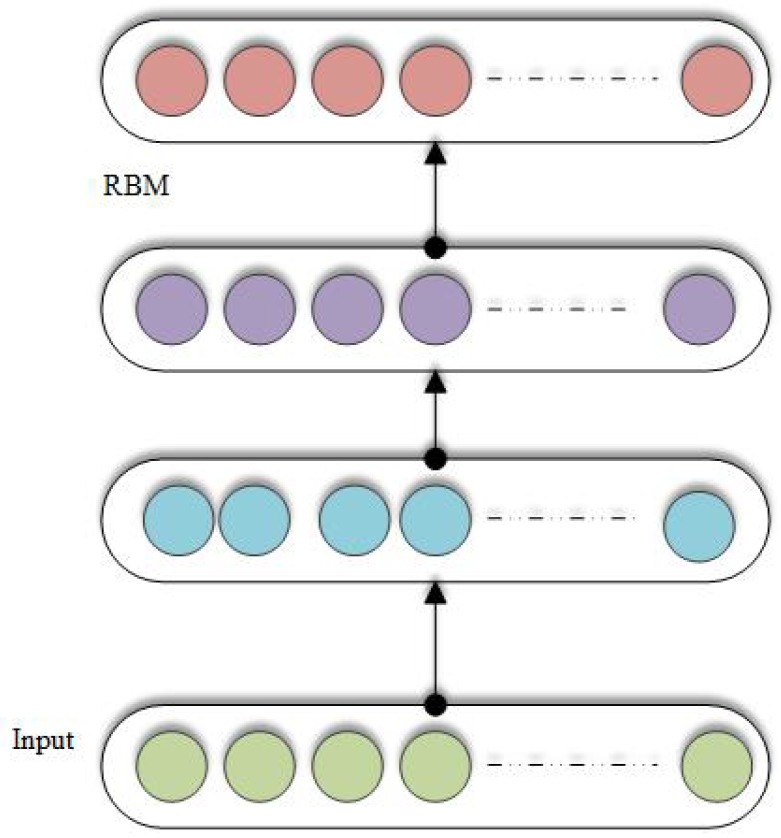
Deep belief networks (DBNs).

Using the stack of Restricted Boltzmann Machines (RBM) [134], a probabilistic generative model is constructed, which is named as Deep belief network (DBN) given in Figure 6. There are two layers of RBN, a visible layer and a hidden layer. Energy function used by RBN is defined as:

(13) $$E\left(u,J\right)=a^{T}u-b^{T}J-u^{T}YJ$$

where *a* is the bias vector of visible layer and *b* is the bias vector of hidden layer. The product of probability of the visible vectors are maximized by the RBM using the following energy function:

(14) $$\arg\max_{Y,a}P\left(u\right)=\frac{1}{Z}\sum_{J}\exp\left(-E(u,J)\right)$$

where *Z* is the partition function. RBM is optimized using the contrastive divergence theorem, which basically combines the Gibbs sampling and gradient descent [135]. DBN is also trained in the greedy fashion. AEs and RBMs share a similar structure.

### 3.13. Adaptive Fuzzy Inference Neural Network (AFINN)

The inference ability of fuzzy, human knowledge expertise and adaptive learning of neural network are combined into a particular machine learning approach known as adaptive fuzzy inference neural network (AFINN). This is a more powerful approach than those based on neural networks or fuzzy logic alone. Sometimes the information gain method is used for the reduction of a number of inputs in AFINN systems. It consists of two layers. One is the input–output (I/O) layer and the other is the rule-layer. The I/O layer consists of the input-part and the output-part. Each node in the rule-layer represents one fuzzy rule. Weights from the rule-layer to the output-part are fully connected and they store fuzzy if-then rules. At learning, stage membership function is automatically tuned. Weights are adjusted in AFINN by backpropagation.

## 4. Evaluation Metrics

True positive (TP) is the correct classification of the positive class, for example if an image contains cancerous cells and the model segments the cancer part successfully and the outcome classifies the presence of cancer. True negative (TN) is the correct classification of the negative class, for example there is no cancer present in the image and the model after classification declares that the cancer is not present. False positive (FP) is the incorrect prediction of the positives, for example the image does have cancerous cells but the model classifies that the image does not contain cancer in it. False negative (FN) is the incorrect prediction of the negatives, for example there is no cancer in the image but the model says an image is a cancerous one.

### 4.1. Receiver Operating Characteristic Curve (ROC-Curve)

The receiver operating characteristic curve (ROC-curve) represents the performance of the proposed model at all classification thresholds. It is the graph of true positive rate vs. false positive rate (TPR vs. FPR).

(15) $$TPR=\frac{TP}{TP+FN}$$

(16) $$FPR=\frac{FP}{FP+TN}$$

### 4.2. Area under the ROC Curve (AUC)

AUC provides the area under the ROC-curve integrated from (0, 0) to (1, 1). It gives the aggregate measure of all possible classification thresholds. AUC has a range from 0 to 1. A 100% correct classified version will have the AUC value 1.0 and it will be 0.0 if there is a 100% wrong classification. It is attractive for two reasons: first, it is scale-invariant, which means it checks how well the model is predicted rather than checking the absolute values; and, second, it is classification threshold invariant as it will check the model’s performance irrespective of the threshold being chosen.

### 4.3. F1-Score

Precision: It checks how precise the model works by checking the correct true positives from the predicted ones.

(17) $$Precision=\frac{TP}{TP+FP}$$

Recall: It calculates how many actual true positives the model hase captured, labeling them as positives.

(18) $$Recall=\frac{TP}{TP+FN}$$

F1-score is the function of precision and recall. It is calculated when a balance between precision and recall is needed.

(19) $$F1=2\times \frac{Precision\times Recall}{Precision+Recall}$$

### 4.4. Accuracy

Accuracy determines that how many true positives *TP*, True negatives *TN*, False positive *FP* and False negatives *FN* were correctly classified:

(20) $$Acc.=\frac{TP+TN}{TP+TN+FN+FP}$$

### 4.5. Specificity

It is the rate of correct identification of negative items:

(21) $$Spef.=\frac{TN}{TN+FN}$$

### 4.6. Sensitivity

Sensitivity is the amount of positive items correctly identified:

(22) $$Sens.=\frac{TP}{TN+FN}$$

### 4.7. Precision

It is the ratio of correctly predicted positive items to the total predicted items:

(23) $$Prec.=\frac{TP}{TP+FP}$$

### 4.8. Jaccard Index

It is a measure of similarity rate between two sample sets:

(24) $$Jac_{idx}.=\frac{TP}{TP+FP+FN}$$

### 4.9. Dice-Coefficient

It is a statistical measure of similarity rate between two sample sets:

(25) $$Dice_{cof}.=\frac{2\times TP}{2\times TP+FP+FN}$$

### 4.10. Average Accuracy

It is a measure of effectiveness of the classifier:

(26) $$Avg_{acc.}.=\frac{\sum_{s=1}^{m}\frac{TP+TN}{TP+TN+FP+FN}}{m}$$

where *m* is the total number outputs of the system.

## 5. Models and Algorithms

### 5.1. Breast Cancer

Much research has already been done for the detection and diagnosis of breast cancer in the past few years. Some of the related papers are briefly discussed here in this section. Albayrak et al. [52] designed a method based on deep learning for the extraction of features applied on histopathological images of the breast, in particular, focused on the detection of mitosis. The proposed model extracted the features from CNN which were fed to support vector machine for its training and the mitosis of the breast was detected. AlexNet was used for the construction of CNN to classify the benign mitosis from the malignant one using the histopathological images [49]. A deep cascade network was proposed for the detection of mitosis from the breast histology slides [85]. From the histology slides, the mitosis candidates were extracted using the trained FCN model. Then, a CaffeNet model [114] was finely tuned and pretrained from the ImageNet images for the mitosis classification. Then, three networks with fully connected layers but different configurations were trained and the outputs were generated in the form of multiple scores or probabilities. These scores were averaged and the final output was generated. In the biomedical context, deep CNNs were explored by Albarqouni and his fellow researchers for non-expert crowd annotations. They proposed a multi-scale CNN architecture in which CNN was combined with the crowd annotations, in such a way that after every softmax layer they introduced an aggression layer (AL) to aggregate the prediction results from multiple participants with annotation results. Classification of nuclei from breast histopathological images using a stacked sparse autoencoder (SSAE) based algorithm was presented in [24]. Optimization of SSAE was done using the greedy strategy, where only one hidden layer at a time was trained and the previous layer’s output becomes the input of the forthcoming hidden layer. In addition to the histopathological images based detection of breast cancer, another dataset was also used in the studies for breast cancer detection that included mammographic images. A hybrid model by combining a CNN along with SVM was introduced in [50] for mass detection on digital mammograms. Mammogram patches were used for the training of the CNN model and high-level feature representation was obtained from the output of the last fully connected layer. This high-level feature representation was used to train the SVM for the classification. In [54], a transfer learning strategy was employed to train a CNN model as the training data were insufficient for training. Using this CNN, it was possible to detect the mass from the available mammograms. When the training data is limited to a few patterns over-fitting can occur. To overcome it, Swiderski et al. presented a way to enrich the training data using a non-negative matrix factorization (NMF) and statistical self-similarity [53]. Ertosun et al. presented a model which at first detects the presence of the mass in the mammograms and then it locates the mass from the mammographic images [51]. To learn the features form mammograms in the multiple scales, Kallenberg et al. trained a model with stacked convolutional sparse autoencoder (SCAE) [136]. The robustness of the model was enhanced by considering a sparsity regularizer in the proposed model. Different potential functions were combined by Dhungel et al. by using a structured support vector machine [137]. These potential functions included the Gaussian mixture model, prior to location, and a deep belief network for the mass segmentation in the mammograms. Dhungel et al. proposed another model using the cascade of random forest classifiers and deep learning for mass detection in another paper [138]. A 3D multi-view model for the learning of bilateral features from digital breast tomosynthesis (DBT) was proposed in [88]. From the source volume, they obtained the volume of interest (VOI), which was treated as a separate input than the VOI in the registered target. To extract high-level features from these two separate VOIs, two separate CNNs were used.

### 5.2. Lung Cancer

In addition to breast cancer, deep learning has found its use in lung cancer as well. Some of the studies which have applied deep learning for this purposed are discussed in this section. Patients survival time was successfully predicted using deep convolutional neural networks by Zhu et al. directly from the lung cancer pathological images [93]. A pretrained CNN, which was trained on a large scale data, was adopted by Paul et al., for the detection of lung cancer by extracting features from the CT images [87]. On the raw images of the lung, the DBN and CNN were applied with end-to-end learning [13]. They used 2D CT images for pulmonary node classifications, whereas, in [61], researchers used 3D CT images on multi-view CNN, which were could be used for end-to-end training. They extracted the 2D patches from the 3D images and used them on CNN for feature extraction. The features were fed to the classifier after fusing them together. As observed in the research study by Dou et al., they formed a model with CNN, which dealt with 3D images directly instead of mapping them into a 2D model [56].

A multi-variant Convolutional neural network (Mc-CNN) was constructed [57]. This model was designed to overcome the problem of variable nodule size. It produces the multi-scale features by replacing the max-pooling layer with the multi-crop pooling layer in the CNN structure. For the nonlinear transform, a randomized leaky rectified linear units (RReLU) was used. Convolutional operation is defined as follows,

(27) $$y^{l}=RReLU\left(\sum_{k}c^{kl}\times h^{k}+b^{l}\right)$$

where $h^{k}$ is the *k*th input map and $y^{l}$ is *l*th output map. $c^{kl}$ are the convolutional kernels between the *k*th input map and *l*th output map. $b^{l}$ is the bias of the lth output map. There were 64 CT slices and so the output features maps were also 64. RReLU is defined as:

(28) $$RReLU=\left\{\begin{array}{cc} x & \mathrm{if}\;x\geq 0\\ \frac{x}{a} & \mathrm{if}\;x<0,a∼U(b_{l},b_{u}) \end{array}\right.$$

where $U(b_{l},b_{u})$ is the uniform distribution and *a* is a random factor sampled from this distribution. $b_{l}$ is the lower bound of the distribution and $b_{u}$ is the upper bound of the distribution. The used max-pooling is defined as:

(29) $$y_{\left(j,k\right)}^{i}=\max_{0\leq m,n<s}h_{\left(j-s+m,k-s+m\right)}^{i}$$

where $y_{\left(j,k\right)}^{i}$ and $h_{\left(j-s+m,k-s+m\right)}^{i}$ are the neuron’s position at (j,k) and (j−s+m,k−s+m) in the *i*th output, respectively, and m and n are the position offsets, whereas *s* is the pool size. Multi-crop pooling strategy is able to capture nodule centric visual features, whereas the traditional max pool is used for the feature subset selection and feature map size reduction. Thus, it can be said that the pooling operation is basically a one-level reduction of the features. In the multi-crop pooling, repetitive pooling strategy is used, which enables the system to obtain multi-scale features. Consider the three concatenated nodule-centric features $f=[f_{0},f_{1},f_{2}]$ formed from $R_{0},R_{1}$ and $R_{2}$ respectively. The size of $R_{0}$ is l×l×n, $R_{1}$ has the size l/2×l/2×n and $R_{2}$ has the size l/4×l/4×n. *n* is the number of features.

(30) $$f_{i}=max-pool^{\left(2-i\right)}R_{i},i=0,1,2$$

max−pool tells the frequency of the max pooling used on the regions $R_{i}$. $R_{1}$ is the center region cropped from the $R_{0}$ thus it is called one time for max pool to generate the feature $f_{0}$. $R_{0}$ is max-pooled twice and generates the feature $f_{1}$. $R_{2}$ is the center region cropped from $R_{1}$; it is not max-pooled but it serves as feature $f_{2}$. The final result of multi-crop would be the concatenation of these features. Minimization of the entropy is done for the learning of this network and is defined as:

(31) $$LOSS=-(qlogP_{1}+\left(1-q\right)logp_{0})$$

where *q* has the suspiciousness value of 1 for high suspiciousness and 0 for the low suspiciousness. Stochastic Gradient descent is followed for the training of the network. The dataset used comprises 1010 patients with the nodule diameter ranging from 3 mm to 30 mm. They achieved an accuracy of 87.14%, sensitivity of 0.77% and specificity of 0.93%.

According to the research carried out by Wang et al., the deep model implementation on lung cancer classification can capture additional information with respect to considering only lung nodules, the information of interest [12]. To avoid this extra information, they calculated 26 handcrafted features and fused them with the CNN extracted features for lung nodules detection [12]. Ground glass opacity (GGO) candidate region selection was made by Hirayama et al. using the fine-tuned CNN model instead of using a pre-trained CNN [59]. GGO candidate regions were calculated by the equation:

(32) $$g\left(x,y,z\right)=\sqrt{{\left(\frac{\Delta \rho x}{\Delta x}\right)}^{2}+(\frac{\Delta \rho y}{\Delta y}{{)}^{2}+(\frac{\Delta \rho z}{\Delta z})}^{2}}$$

where *x*, *y* and *z* directions were determined by the equations:

(33) Δρx=|ρ(x+1,y,z)−ρ(x,y,z)|+|ρ(x,y,z)−ρ(x−1,y,z)|

(34) Δρx=|ρ(x,y+1,z)−ρ(x,y,z)|+|ρ(x,y,z)−ρ(x,y−1,z)|

(35) Δρx=|ρ(x,y,z+1)−ρ(x,y,z)|+|ρ(x,y,z)−ρ(x,y,z−1)|

The morphological opening was performed followed by the labeling techniques and the noise was reduced using thresholding methods for each label volume sphericity. This process generated the GGO candidates at the end. They used the support vector machine classifier and achieved 93% true positives and 210.52% false positives.

### 5.3. Brain Cancer

Brain cancer has an uncontrolled growth and it may occur in any part of the brain. It has been quite challenging to detect which part of the brain contains cancer. Consequently, the biggest challenge for brain cancer is the segmentation of the brain from the healthy part. Several challenges have been conducted by BRATS for this purpose. Here, we include some of the research work in which deep learning has been successfully applied to brain images. Two algorithms based on 2D CNN and 3D CNN were proposed by Gao et al., working on 2D sliced images and 3D images, respectively. The final result was obtained by fusing the output from these two models. This hybrid model outperformed the 2D and 3D scale-invariant features swift (SIFT) and Kaze features. The automatic magnetic resonant image segmentation method based on CNN was discussed by Author1 [80]. They investigated the intensity normalization and augmentation for brain tumor detection. By exploring the local and global contextual features in the CNN model, Havaei et al. used a fully connected layer in the final layer of CNN to increase the speed of the system and detected the brain cancer successfully. A fully connected convolutional neural network (FCN) and conditional random field (CRFs) were used in [79] for the brain cancer segmentation. First, the image patches were used to train the FCN model and the training of CRF was done. In the end, the system was finely tuned using the image slices directly. Adjacent image patches were joined together into one pass using a dense training scheme in the CNN model [81]. The false positives were removed by using the 3D fully connected random field, after the 3D segmentation of the images using modality of CNN. Zhao et al. combined the multi-modality information from T1, T1C, T2, and fluid-attenuated inversion recovery (FLAIR) images and trained the proposed CNN from this information [82]. The algorithm proposed by them was a 3D voxel classification based on CNN. Different scaled 2D patches were extracted from 2D slices obtained by slicing the 3D dataset and these 2D patches were fed to multiple CNNs for the learning process.

### 5.4. Skin Cancer

“Melanoma” is curable if it is detected early. Differentiating between benign melanoma and malignant melanoma is really difficult, as they appear to be the same in the early stages. The main causes of melanoma and the risk factors are provided in Table 6 [139]. Many methods have been used to differentiate among them, including the most famous ABCD rule, seven-point checklist method, Menzies method, and pattern analysis.

Pomponiu et al. used 399 images captured from the standard camera to classify benign nevi from melanoma [65]. Firstly, preprocessing was performed along with data augmentation. High-level features of skin samples were extracted using pre-trained CNN and AlexNet. For lesion classification, K-nearest neighbor was used. They were able to achieve the accuracy of 93.62% with a specificity of 95.18% and sensitivity rate of 92.1%. In total, 129,450 images were used by Esteva et al. for the pretraining of CNN, 2032 of which were from a skin lesion and the rest were taken from dermatoscopic devices [86]. There were two types of classifications: (1) benign nevi’s classification from malignant melanoma; and (2) benign seborrheic keratosis classification from keratinocytes carcinomas. They used transfer learning for the classification. The AUC achieved was 0.96 for both melanomas and carcinomas. A pre-trained CNN along with pre-trained AlexNet and VGG-16 [140] were used for deep feature extraction and lesion classification [62]. Using 19,398 images for training a ResNet model, Han et al. proposed a classifier model for classifying 12 different types of skin diseases [13]. With the help of the Asan dataset, the achieved AUCs were 0.83 for squamous cell carcinoma, 0.82 for intraepithelial carcinoma, and 0.96 for melanoma and basal cell carcinoma. Despite the presence of pre-trained CNN’s, some efforts were made to develop new CNN algorithms. A self-advised semi-supervised learning model was proposed by massod et al. [141], for melanoma detection. Their proposed system consisted of a deep belief network and two self-advised support vector machines (SA-SVM) trained on three different datasets, along with two kernels radial basis function kernel (RBF) and polynomial kernel, respectively. The maximization of labeled data separation was done by a fine-tuning procedure with an exponential loss function. Deep features and hand-crafted features were combined in [67]. In the proposed system there were two SVM classifiers, one trained on local binary patterns (LBPs) and rotated speeded-up robust features (RSurf), while the other was trained on raw color images using the deep features extracted by the CNN model and probability scores were generated. The final decision was based on higher scores. Sabbaghi et al. used deep neural networks and mapped the images to enhance classification accuracy into bag-of-feature (BoF) space [167]. Demyanov et al. used stochastic gradient descent to train the CNN model and detect the typical network patterns and regular globules patterns in [68]. Yu et al. used residual blocks to replace FCN’s convolutional layers and therefore formed a fully convolutional residual network (FCRN), which is further used for the classification purpose [142]. Nasr-Esfahani et al. detected melanoma by feeding preprocessed images to CNN model [70], whereas border detection based CNN system was proposed in [74] for skin cancer diagnosis. Author1 [143] and Author1 [42] used the ABCDE method for cancer detection along with the image processing tools of segmentation, histogram analysis, and contour tracing. Sujaya et al. studied lesion probability using graphical user interface [144]. Palak et al. proposed a method using Fuzzy C Mean (FCM) for skin cancer analysis [145]. Colored Unsupervised segmentation, k means clustering and Gradient Vector Flow (GVF) were used in [146]. Sumithra et al. used Support Vector Machine (SVM) and K-nearest neighbors (K-NN) for lesion analysis [147]. In this section, the algorithms proposed in 2018 for skin cancer detection are briefly summarized. Jianfeng et al. used a backward-propagation method in a eight-layer CNN model [148]. Nine hundred images were used for classification testing. The achieved performance were 91.92% and 89.5% accuracy on the training set and test set, respectively. A system based on content-based image retrieval CBIR was proposed by P. Tschandi et al. in comparison with CNN. Three datasets were used to train the neural network, including 888, 2750 and 16,691 images. The prediction was done using Softmax. Performance measures were area under the characteristic-curve (AUC) and mAP (multi-class-accuracy and mean-average-prediction). Dataset 1 achieved 0.842 AUC value and 0.830 mAP value; Dataset 2 achieved 0.806 AUC value and 0.810 mAP value; and Dataset 3 achieved 0.852 AUC value and 0.847 mAP value. This was further tested on eight classes and performed well on that as well with respect to the normal CNN model. The dataset provided by ISIC in the 2016 challenge was used in [149] for the classification of the lesion using the CNN model along with ANN. Firstly, image segmentation was performed using intensity thresholding and then they used CNN for feature extraction. ANN classifier used these features to perform the classification. According to them, they achieved 98.32% accuracy, which was better than the previous best 97%. T.C. Pham et al. proposed a method for improvement of classification using CNN along with the method of data-augmentation [150]. In addition, they tried to overcome the issue of data limitation and its influence on the classifier’s performance. The dataset used contained 600 images for testing and 6162 for training. AUC value achieved was 89.2%, ACC value was 89.0% and AP value was 73.9%. They studied the influence of image augmentation on three different classifiers and found that they performed differently and showed improved results compared to the standard methods used previously. Four cutaneous diseases were diagnosed by using deep learning methods [151]. They made a hierarchical structure to make a summary of classification and diagnosis criteria. They were able to achieve the accuracy of 87.25% with a probability error of 2.24%. A SkinNet convolutional neural network was used in [152] for the segmentation and detection of skin cancer. A modified version of U-net CNN was proposed. A comparison of their results was made with state-of-the-art techniques. The dataset used in this work was from the 2017 ISBI challenge. They achieved the average value of the dice-coefficient of 85.10%, Jaccard index of 76.67% and sensitivity evaluation of 93.0%. H.A. Haenssle used 100 test images on google inception V4 CNN model in two levels [153]. At first, only dermoscopic images were used and then clinical images were also used along with dermoscopic images. Comparison was done with the 58 dermatologists internationally as well as with the five algorithms from 2016 ISBI challenge. Level 1 achieved the sensitivity of 86.6% with a standard deviation of 9.3%, and a specificity of 71.3% with a standard deviation of 11.2%. Level 2, where clinical images were also added, improved results to the sensitivity rate of 88.9% with a standard deviation of 9.6% and specificity of 75.7% with a standard deviation of 11.7%. Y. Wang used DeepLab 3, where instead of convolutional neural networks, they proposed atrous convolution method for segmentation of input image [154]. They achieved the Jaccard index of 0.498. Further improvement is necessary to improve the performance of the system. Different methods were tested on the vector extracted by PH2 using a dataset of dermatoscopic images [155]. Overall, 92.5% accuracy and 85.71% precision using Logistic Regression and VGG19 network was achieved. A multi-task convolutional neural network with the framework of joint detection and segmentation called faster region-based CNN was proposed in [156]. Region proposals and bounding boxes were generated by region proposal network (RPN) for localization of the lesion. Softmax refines these bounding blocks, which were then further processed for cropping, and SkinNet was used for their segmentation. Using the dataset of the 2017 ISBI challenge, this method achieved a dice coefficient of 0.93, accuracy of 0.96, Jaccard index of 0.88 and sensitivity of 0.95. A. Rezvantalab et al. used multiple state-of-the-art architectures to classify eight types of skin diseases [157]. The dataset contained 10,135 images including melanoma, nevi, BCC, BK, AK, ITC, DF, vascular lesion and atypical nevi. The architectures used were ResNet 152, Inception ResNet v2, Inception v3, and DenseNet 201. The AUC of ResNet 152 for melanoma and BCC classification was 94.40% and for DenseNet its value was 99.30%, while the average AUC value by DenseNet 201 was 98.16%. In an article published in JAMA Dermatology in January 2019, Philipp et al. used combined CNNs for pigmented melanocyte lesions to achieve expert-level accuracy. Their dataset consisted of 13,724 images, 7895 of which were dermoscopic images and 5829 were closeup images. The data were collected from 2008 to 2017 of lesions at a skin cancer clinic. Testing of this algorithm was made in 2072 cases, while the comparison was made by 95 medical experts (Human raters) and 62 board-certified dermatologists. They observed that cCNNs performed well as compared to human raters and they achieved a high percentage of correct diagnosis. Walker et al. used deep learning to improve the diagnosis of skin lesion [158]. They conducted two levels of study, one of which called LABS (Laboratory retrospective study) and the second one non-interventional OBS (Observational study). For experimenting with LABS, 482 biopsies were used while 63 biopsies were used for OBS. A deep learning classifier was trained on 3954 training visual data. Then, on the output of this classifier, sonification was performed (which means the conversion of the signal into sound files). Then, there was a second ML classifier that operates this raw sound for LABS and image analysis for OBS. This algorithm provided AUC of 0.976; AUC for raw sound was 0.931, for FFT was 0.90 and for spectrogram was 0.988. OBS obtained AUC of 0.819 from raw sound and 0.808 from image analysis. They proved that the addition of the second stage on the DL algorithm including sonification and heuristic analysis can improve accurate diagnosis. A new cause of cancer found in 2018 by Zioutas and Valachovic was counter argued by Hector this year. According to them, there is a connection between melanoma cancer and dark matter. They proposed the idea that inner planetary motion significantly increases the dark matter density on Earth. They specifically considered the planetary motion of Mercury and Earth. According to them, this increase in density of dark matter causes melanoma cancer, to which the black population is pretty much immune. To counter their argument, Hector used the same large amount of data and also presented periodic stats provided by Zioutas and Valachovic. Yoshimasa et al. used a convolutional neural network for the detection of esophageal cancer, SCC (Squamous cell carcinoma), and adenocarcinoma [159]. The training images used in this study included 8428 images collected from 384 patients in Japan. Test data contained 1118 images where 47 patients had esophageal cancer while 50 did not. They were able to achieve the accuracy of 98% while the sensitivity achieved was 98%. Forty percent of each image was predicted positively while 95% was negatively predicted because of the shadows, which was the reason for misdiagnosis. Gomez-Martin et al. studied clinical, dermoscopic and confocal parameters for the detection of flat leg lesions pink shaded in elders [160]. They achieved the accuracy of 49.1%, the specificity of 73.4% and the sensitivity of 68.7% with the clinical diagnosis system. While dermoscopy provided 59.6% accuracy, and 85% and 67.6% sensitivity and specificitym. respectively. Confocal microscopy achieved accuracy of 85.1%, and 97.5% and 88.2% sensitivity and specificity, respectively. Parpti et al. used image enhancement to improve the image quality followed by the multi-scale retinex MSR along with Color restoration to detect skin cancer [161].

### 5.5. Prostate Cancer

Prostate cancer is the third highest cause of death among men and it has a high chance of diagnosing in males [162]. To facilitate timely radiotherapy, its successful segmentation is very important. A combination of sparse patch matching and deep feature learning for prostate segmentation was proposed by Author1 [162]. To extract the feature representation from the MR images, they used the SSAE technique. Author1 [76] proposed a prostate cancer detection method using the SAE classifier (Table 7).

The collected features were improved by the supervised way fine-tuning of the SSAE model. The recognition map was refined by using the energy minimization procedure based on the neighbor pixel relationship. Tian et al. used a fully convolutional network for prostate segmentation [165]. Using the 3D MR images, Yu et al. segmented the prostate by using volumetric convolutional networks [142]. They extended their FCN with residual blocks to enable the volume to volume prediction. Maa et al. Proposed a method using patch-based CNN to use the region of interest and detect prostate cancer from its [77]. The final segmentation result was obtained by using a multi-atlas label fusion. Lumen segmentation using the CNN model was done in [92] and for the classification of prostate cancers, they generated maps. Patch-based CNN was used in [90].

## 6. Discussion

According to the reviewed studies, CNN has the best in performance of all architectures. The winner of ImageNet Large Scale Visual Recognition Competition (ILSVRC) 1998 was LeNet, which is a seven-level CNN architecture, and 2012 it was AlexNet, which is also a very successful version of CNN. From 2012 to 2015, the winner of this competition has been the CNN architectures AlexNet, ZFNet, GoogleNet/ Inception V1, VGGNet and ResNet, which shows the success rate of the CNN architectures in this field. Since these are all different architectures of the same CNN, as the model changes, the only evaluation measure is their percentage performance. As described in the competition, the necessary part was the reduction of top-5 errors, which AlexNet reduced from 26% to 15.3%, while ZFNet reduced to 14.8%. This performance was beaten by the GoogleNet/Inception V4, achieving the error reduction to 3.6%. The best performance was shown by ResNet, which beats the human-level performance by reducing errors to 3.57%.

When implementing deep learning for cancer diagnosis, one of the major challenges becomes a lack of availability of datasets. Every learning algorithm requires a large amount of training for performance measure. However, efforts have been made to make medical images archives containing confidential information of many patients by picture archiving and communication society (PACS). Researchers also use data images from cancer research organizations and hospitals for executing their algorithms. One of the major breakthroughs for data collection was made by Esteva et al. [86]. They collectively made an effort and formed a dataset with 127,463 training images and 1942 test images. Many researchers use a small dataset for their algorithms. In addition, most of the datasets available online with open access have raw images and so researchers are required to obtain the ground truth themselves.

To deal with the issue of limited dataset, a scheme of data augmentation was proposed. Many researchers use data augmentation, which includes techniques such as rotation, cropping and filtering to increase the number of available data. Another way to avoid over-fitting is transfer learning, which has been used by many of the researchers discussed above in this review.

Low contrast and SNR of medical images are responsible for the poor performance of deep learning algorithms. Thus, another issue is how to improve the performance of the proposed model if the data have low contrast and poor SNR. Furthermore, studies based on brain tumor segmentation raised a question: How can we maintain the performance of algorithms on multiple resource data When the algorithms were made to train on multi-institutional data, their performance decreases gradually. Some of the online available datasets along with their access links are given in Table 8.

Another issue that was observed is the inequality of training data distribution. If the positive data are made larger than the negative data, then the system will be automatically biased and will majorly give positive results while the same happens if the data have more negative than positive cases. Thus, equality of training data is very important, which was ignored by few researchers.

One of the problems faced when implementing a convolutional neural network is the size of the target object inside the image. As the target object varies in size, studies proposed to train the model with images of various scales to make the model learn this size variation. To capture the multi-scale features directly from image, Shen et al. replaced a standard pooling operation with multi-crop pooling [57].

## 7. Summary and Conclusions

This review focuses on providing all the necessary information to the beginners of this field, starting from the main concepts of cancer diagnosis, evaluation criterion and medical methods. As this manuscript mainly focuses on the deep learning for cancer diagnosis, the most important things to introduce to our readers are all the possible techniques of deep learning that can be used for diagnostic purposes in this document. Furthermore, to facilitate the audience, the respective practice codes for each technique, which are easily available online, are put together in a table. One of the major issues that one can encounter in implementing any algorithm is the dataset availability, therefore all possible access links to the datasets are presented in this work.

Different architectures of CNN are also described in this manuscript. The implementation of the deep learning algorithms for brain cancer, lung cancer, breast cancer, and skin cancer is the focus of this manuscript. The performance measures for different studies are provided. In this review, different deep learning algorithms for classifying different types of cancers are presented. In this review, fifteen studies used Histopath model with CNN for classification and detection of different types of cancers as provided in Table 9. Six of these studies provided the source of data [49,50,52,54,85] while nine studies did not publish the source of data [90,91,92,93,95,101,102,104]. Two research studies used mammographs for detection along with CNN and published data source [51,53]. Eight studies [12,59,77,87,96,98,103,105] used CT Slices, three of which used data from PROMISE [75], and LIDC [166]. Five studies used volumetric computed tomography [56,57,58,60,61]. Seven studies were for brain cancer classification [76,79,80,81,89,94,94].

In the field of dermatology, Esteva et al. used pre-trained CNN for skin lesion classification with accuracy of 93.62% [86]. Sabbaghi et al. mapped images to bag-of-feature to increase classification accuracy [167]. Globule patterns on the skin were detected by Demyanov et al. using a stochastic gradient descent model [68]. Yu et al. formed FRCN by replacing the FCN’s Conv. layer with the residual layer [142]. Melanoma detection was performed by Nasr et al. by feeding preprocessed images to CNN network model [70].

Two of the methods reviewed in this study by Chandrahasa et al. and M.Garbaj et al. used the ABCDE method for skin cancer detection; they made use of image segmentation, histogram analysis and contour tracing [42,143]. Sujaya et al. used a graphical user interface to classify skin lesion [144]. whereas fuzzy C Mean was used by Palak et al. for skin cancer analysis [145]. Sumithra et al. used support vector machine for skin lesion classification [147].

In total, 27 different algorithms provided by 27 different researchers [42,65,67,68,70,74,86,141,142,143,144,145,147,148,149,150,151,152,153,155,156,157,158,159,161,167] are reviewed for skin cancer diagnosis. As discussed above, there are different methods with different algorithm schemes and different training datasets, which adds difficulty when comparing them. No particular standard can be defined to compare their results. 

## Figures and Tables

**Table 1:** Steps of cancer diagnosis.

Pre-Processing	Image Segmentation	Post-Processing
Contrast adjustment	Histogram thresholding	Opening and closing operations
Vignetting effect removal	Distributed and localized	Island removal
	Region identification	
Color correction	Clustering & Active contours	Region merging
Image smoothing	Supervised learning	Border expansion
Hair removal	Edge detection & Fuzzy logic	Smoothing
Normalization and localization	Probabilistic modeling and graph theory

**Table 2:** Menzies method.

Pos.F	Neg.F
Blue-white veil	Lesion’s symmetry
Depegmentation like scars	Single color presence
Multi-colors	
Gray and blue dots	
Broadened networks	
Psuedopods	
Globules	
Radial streaming	

**Table 3:** List of available codes online.

Method Name	Online Code Link
Convolutional Autoencoder	https://sefiks.com/2018/03/23/convolutional-autoencoder-clustering-images-with-neural-networks/
Stacked Autoencoder	https://github.com/siddharth-agrawal/Stacked-Autoencoder
Restricted Boltzmann Machine	https://github.com/echen/restricted-boltzmann-machines
Recurrent neural networks	https://github.com/anujdutt9/RecurrentNeuralNetwork
Convolutional	https://gist.github.com/JiaxiangZheng/a60cc8fe1bf6e20c1a41abc98131d518
Neural networks	https://github.com/siddharth-agrawal/Convolutional-Neural-Network
Multi-scale CNN	https://github.com/alexhagiopol/multiscale-CNN-classifier
Multi-instance	https://github.com/AMLab-Amsterdam/AttentionDeepMIL
Learning CNN	https://github.com/yanyongluan/MINNs
Long short-term memory	https://github.com/wojzaremba/lstm

**Table 4:** Summary of references with CNN Applications. (Histo.path: histogram pathology, vol.CT: Volumetric computed, MG: Mammographs, MRI: Magnetic Resonance Imaging, DermoS: Dermoscopic Segmentation, and BraTS: Brain tumor segmentation.)

Application Type	Modality	Dataset	Reference
Breast Cancer Classification	Histo.path	BreakHis	Spanhol et al. [49]
Mass Detection	Histo.path	INbreast	Wichakam et al. [50]
Mass segmentation	Mammo.graph.	DDSM	Ertosun et al. [51]
Mitosis Detection	Histo.path	MITOSATYPIA-14	Albayrak et al. [52]
Lesion recognition	Mammo.graph.	DDSM	Swiderski et al. [53]
Mass Detection	Histo.path	DDSM	Suzuki et al. [54]
Lung nodule (LN) Classification	CT Slices	JSRT	Wang et al. [55]
Pulmonary nodule Detection	Volumetric CT	LIDC-IDRI	Dou et al. [56]
Lung nodule (LN) Suspiciousness classification	Volumetric CT	LIDC-IDRI	Shen et al. [57]
Nodule characterization	Volumetric CT	LIDC-IDRI	Hua et al. [58]
Ground glass opacity (GCO) extraction	CT Slices	LIDC	Hirayama et al. [59]
Pulmonary nodules detect.	Volumetric CT	LIDC	Setio et al. [60]
Nodule Characterization	Volumetric CT	DLCST, LIDC, NODE09	Hussein et al. [61]
Skin lesion classification	Dermo.S.	ISIC	Mahbod et al. [62]
Skin lesion classification	Dermo.S.	DermIS, DermQuest [63,64]	Pomponiu et al. [65]
Skin lesion classification	Dermo.S.	ISIC [66]	Majtner et al. [67]
Dermoscopy patterns classification	Dermo.S.	ISIC	Demyanov et al. [68]
Melanoma detection	Clinical photoghrapy	MED-NODE [69]	Nasr-Esfahani et al. [70]
Lesion border detection	Clinical photoghrapy	DermIS, Online dataset, DermQuest [71,72,73]	Sabouri et al. [74]
Prostate Segmentation	MRI	PROMISE12 [75]	Yan et al. [76]
Prostate Segmentation	CT Scans	PROMISE12	Maa et al. [77]
Brain tumor Segmentation Cancer detection	MRI	BraTS [78]	Zhao et al. [79]
Brain tumor Segmentation	MRI	BraTS	Pereira et al. [80]
Brain tumor Segmentation	MRI	BraTS	KAmnitsas et al. [81]
Prostate segmentation	MRI	BraTS	Zhao et al. [82]
Gland segmentation	Histo.path	Warwick-QU [83,84]	Chen et al. [85]

**Table 5:** Summary of references with CNN Applications.

Application Type	Modality	Reference
Dermatologists-level skin cancer	Dermo.S.	Esteva et al. [86]
Survival Prediction	CT Slices	Paul et al. [87]
Latent bi[]=lateral feature representation learning	Tomosynthesis	Kim et al. [88]
Feature learning of Brain tumor	MRI	Liu et al. [89]
Gleason grading	Histo.path	Kallen et al. [90]
Gleason grading	Histo.path	Gummeson et al. [91]
Lumen-based Prostate	Histo.path	Kwak et al. [92]
Survival analysis	Histo.path	Zhu et al. [93]
Classification of Brain tumor	MRI	Ahmed et al. [94]
Cervical cytoplasm and nuclei segmentation	Histo.path	Song et al. [95]
Urinary bladder	CT Slices segmentation	Cha et al. [96]
Liver segmentation on Laparoscopic videos	Laparoscopy	Gibson et al. [97]
Inner/outer bladder wall segmentation	CT Slices	Gordon et al. [98]
Cervical dysplasia diagnosis	Digital cervicigraphy	Xu et al. [99]
Colon adenocarcinoma glands segmentation	Histo.path	BenTaieb et al. [100]
Nucleus segmentation	Histo.path	Xing et al. [101]
Circulating tumor-cell detection	Histo.path	Mao et al. [102]
Liver tumor segmentation	CT Slices	Li et al. [103]
Cervical cytoplasm segmentation	Histo.path	Song et al. [104]
bladder cancer Treatment response assessment	CT Slices	Cha et al. [105]

**Table 6:** Skin cancer risk factors and its causes.

Cause	Risk Factors
1. Sunlight	(a) UV radiations leading to cancer
	(b) Sunburn Blisters: sunburns in adults are more prone to cancer
	(c) Tanning
2. Tanning Booths	leads to cancer before the age of 30 and Sun lamps
3. Inherited	Two or more careers of melanoma from family inherit this disease to the descendants
4. Easily burnable skin	Gray/Blue eyes, Fair/Pale skin, Blond/Red hairs
5. Medications Side-Effects	Side effects of anti-depressants antibiotics and Hormones

**Table 7:** Summary of CNN for different cancers.

Application Type	Modality	Reference
Prostate Segmentation	3D MRI	Yu et al. [142]
Prostate Segmentation	3D MRI	Milletari et al. [163]
Prostate Segmentation	MRI	Zhao et al. [79]
Polyp detection	clonoscopy	Yu et al. [164]

**Table 8:** Datasets and their online access links.

Dataset Name	Link to Data Access
ISIC	https://challenge.kitware.com/#challenge/5aab46f156357d5e82b00fe5
DermIS	http://biogps.org/dataset/tag/dermis/
BRATS	https://www.med.upenn.edu/sbia/brats2018/data.html
PROMISE-12	https://promise12.grand-challenge.org/Details/
DerQuest	https://www.derm101.com/dermquest/
DLCST	https://clinicaltrials.gov/ct2/show/NCT00496977
MED-NODE	http://www.cs.rug.nl/ imaging/databases/melanoma_naevi/
JSRT	http://db.jsrt.or.jp/eng.php
LIDC	https://wiki.cancerimagingarchive.net/display/Public/LIDC-IDRI#940027f1a8a845d0a61a1b5b5083567e
DDSM	http://www.eng.usf.edu/cvprg/
BreakHis	https://web.inf.ufpr.br/vri/databases/breast-cancer-histopathological-database-breakhis/
INBreast	http://medicalresearch.inescporto.pt/breastresearch/index.php/Get_INbreast_Database
MITOSTAPIA	https://mitos-atypia-14.grand-challenge.org/dataset/
Warwick-QU	https://warwick.ac.uk/fac/sci/dcs/research/tia/glascontest/about/
mini-MIAS	http://peipa.essex.ac.uk/info/mias.html
Proeng	http://visual.ic.uff.br/en/proeng/thiagoelias/
AMD-Retina	https://www.dropbox.com/s/mdx13ya26ut2msx/iChallenge-AMD-Training400.zip?dl=0
PAIP-2019	https://paip2019.grand-challenge.org/Dataset/
KITS	https://kits19.grand-challenge.org/data/
CHAOS	https://chaos.grand-challenge.org/Data/
EAD-2019	https://ead2019.grand-challenge.org/Data//
ANHIR	https://anhir.grand-challenge.org/Data/
ABCD	https://nda.nih.gov/edit_collection.html?id=3104
segTHOR	https://competitions.codalab.org/competitions/21012Clearn_the_details-dataset
segTHOR	https://competitions.codalab.org/competitions/21012#learn_the_details-dataset
Kaggle	https://www.kaggle.com/c/histopathologic-cancer-detection/data
CTC19	http://celltrackingchallenge.net/datasets/
cuRIOUS	https://curious2018.grand-challenge.org/Data/
LUMIC	https://lumic.grand-challenge.org/Dataset/
IDRID	https://idrid.grand-challenge.org/Data/
BACH	https://iciar2018-challenge.grand-challenge.org/Dataset/
RSNA	https://www.kaggle.com/c/rsna-pneumonia-detection-challenge/data
MUSHAC	https://projects.iq.harvard.edu/cdmri2018/data
IVDM3Seg	https://ivdm3seg.weebly.com/data.html
MRBrainS18	https://mrbrains18.isi.uu.nl/data/
18F-FDG PET	https://www.kaggle.com/c/pet-radiomics-challenges/data
BRATS	https://www.med.upenn.edu/sbia/brats2018/data.html
SICAS	https://www.smir.ch/
Bowl	https://www.kaggle.com/c/data-science-bowl-2018/data
TADPOLE	https://tadpole.grand-challenge.org/Data/
CATARACTS	https://cataracts.grand-challenge.org/Data/
RETOUCH	https://retouch.grand-challenge.org/Download/
CAMELYON17	https://camelyon17.grand-challenge.org/Data/
BOWL17	https://www.kaggle.com/c/data-science-bowl-2017/data
ISEG17	http://iseg2017.web.unc.edu/download/
ACDC17	https://www.creatis.insa-lyon.fr/Challenge/acdc/databases.html
ISLES	http://www.isles-challenge.org/ISLES2017/
CCSD	https://www.kaggle.com/c/intel-mobileodt-cervical-cancer-screening/data
LUNA	https://luna16.grand-challenge.org/Data/
AIDA-E	https://isbi-aida.grand-challenge.org/
CAMELYON16	https://camelyon16.grand-challenge.org/Data/
STACOM-SLAWT	http://www.doc.ic.ac.uk/ rkarim/la_lv_framework/wall/datasets.html
SMLM	http://bigwww.epfl.ch/smlm/datasets/index.html
MTOP	https://tbichallenge.wordpress.com/data/
CREMI	https://cremi.org/data/
BOWL!5	https://www.kaggle.com/c/second-annual-data-science-bowl/data
DREAM	https://www.synapse.org/#!Synapse:syn4224222/files/
ISBI15	http://www-o.ntust.edu.tw/ cweiwang/ISBI2015/challenge2/index.html
TRACTOMETER	http://www.tractometer.org/ismrm_2015_challenge/data
CANCER	https://www.cancerimagingarchive.net/#collections-list
BRATS15	https://www.cancerimagingarchive.net/
CLUST	https://clust.ethz.ch/data.html
PDDCA	http://www.imagenglab.com/newsite/pddca/
CETUS	https://www.creatis.insa-lyon.fr/Challenge/CETUS/databases.html
OCCISC-14	https://cs.adelaide.edu.au/ carneiro/isbi14_challenge/dataset.html
CAD-PE	http://www.cad-pe.org/?page_id=12
SMLM-SB	http://bigwww.epfl.ch/smlm/datasets/index.html
HARDI	http://hardi.epfl.ch/static/events/2013_ISBI/

**Table 9:** Summary of references for different cancers.

Application Name	References	No. of Papers
Breast Cancer	[49,50,52,53,54,85,88,114,136,137,138]	12
Lung Cancer	[12,56,57,59,60,61,87,93]	12
Prostate Cancer	[76,77,90,91,92]	5
Brain Cancer	[79,80,81,89,94]	5
Skin Cancer	[42,65,67,68,70,74,86,141,142,143,144,145,147,148,149,150,151,152,153,154,155,156,157,158,159,161,167]	27

## Abbreviations

The following abbreviations are used in this manuscript:

ABCD	Asymmetry, Border, Color Variation and Diameter
ABCDE	Asymmetry, Border, Color Variation, Diameter and Expansion
AD	Adenocarcinoma
ANN	Artificial neural networks
AI-ANN	Artificial intelligence-artificial neural networks
AK	Actinic Keratosis
AUC	Area under the characteristic curve
BoF	Bag-of-features
BCC	Basal cell carcinoma
BK	Benign Keratosis
CAD	Computer aided diagnosis
CAE	Convolutional autoencoders
CNNs	Convolutional neural networks
cCCNs	Combined convolutional networks
CT	Computed tomography
DANs	Deep auto encoders
DF	Dermatofibroma
FCNs	Fully convolutional networks
FCRN	Fully convolutional Residual networks
FCM	Fuzzy-c Mean
FPS	Fourier power spectrum
FP	False positives
FN	False negatives
GVF	Gradient Vector Flow
GAN	Generative adversarial models
GPU	Graphics processing units
GLCM	Grey level co-occurrence matrix
GM	Generalization mean
histo.path	Histopathology
IC	Intraepithelial carcinoma
ILSVRC	ImageNet large scale visual recognition competition
K-NN	K-Nearest neighbor
LBPS	Local binary patterns
LABS	Laboratory retrospective study
LTSM	Long short-term memory
M-CNN	Multi-scale convolutional neural network
MIL-CNN	Multiinstance convolutional neural network
MRI	Magnetic resonance image
mAP	multi class accuracy and mean average prediction
Neg.F	Negative features
OBS	Observational study
PACS	Picture archiving and communication society
Pos.F	Positive features
PCA	Principal component analysis
RBF	Radial basis function
RBM	Restricted Boltzmann’s machine
ReLU	Rectified linear unit
ROC	Receiver operating characteristic curve
ROI	Region of interest
Rsurf	Rotated speeded-up robust features
RNN	Recurrent neural networks
SAE	Stacked autoencoders
SA-SVM	Self-advised support vector machine
SCC	Squamous cell carcinoma
SNR	Signal too noise ratio
SVM	Support Vector Machine
TP	True positives
TN	True negatives
WPT	Wavepacket transform
